# Expression profile of host restriction factors in HIV-1 elite controllers

**DOI:** 10.1186/1742-4690-10-106

**Published:** 2013-10-16

**Authors:** Mohamed Abdel-Mohsen, Rui André Saraiva Raposo, Xutao Deng, Manqing Li, Teri Liegler, Elizabeth Sinclair, Mohamed S Salama, Hussam El-din A Ghanem, Rebecca Hoh, Joseph K Wong, Michael David, Douglas F Nixon, Steven G Deeks, Satish K Pillai

**Affiliations:** 1 Department of Medicine, University of California San Francisco, San Francisco, California, USA; 2 Division of Experimental Medicine, University of California San Francisco, San Francisco, California, USA; 3 Blood Systems Research Institute, San Francisco, California, USA; 4 Division of Biological Sciences, Section of Molecular Biology, University of California San Diego, La Jolla, California, USA; 5 Faculty of Science, Ain Shams University, Cairo, Egypt; 6 Department of Medicine, San Francisco Veterans Affairs Medical Center, San Francisco, California 94118-4417, USA

**Keywords:** Elite controllers, Intrinsic immunity, Retroviral restriction factors, APOBEC3, BST2/tetherin, TRIM, schlafen 11, p21, T cell activation

## Abstract

**Background:**

Several host-encoded antiviral factors suppress HIV-1 replication in a cell-autonomous fashion *in vitro*. The relevance of these defenses to the control of HIV-1 *in vivo* remains to be elucidated. We hypothesized that cellular restriction of HIV-1 replication plays a significant role in the observed suppression of HIV-1 in "elite controllers", individuals who maintain undetectable levels of viremia in the absence of antiretroviral therapy (ART). We comprehensively compared the expression levels of 34 host restriction factors and cellular activation levels in CD4+ T cells and sorted T cell subsets between elite controllers, HIV-1-infected (untreated) non-controllers, ART-suppressed, and uninfected individuals.

**Results:**

Expression of schlafen 11, a codon usage-based inhibitor of HIV-1 protein synthesis, was significantly elevated in CD4+ T cells from elite controllers as compared to both non-controllers (p = 0.048) and ART-suppressed individuals (p = 0.024), with this effect most apparent in central memory CD4+ T cells. Schlafen 11 expression levels were comparable between controllers and uninfected individuals. Cumulative restriction factor expression was positively correlated with CD4+ T cell activation (r^2^ = 0.597, p < 0.0001), viral load (r^2^ = 0.34, p = 0.015), and expression of ISG15 (r^2^ = 0.73, p < 0.0001), a marker of interferon exposure. APOBEC3C, APOBEC3D, CTR9, TRIM26, and TRIM32 were elevated in elite controllers with respect to ART-suppressed individuals, while levels were comparable to uninfected individuals and non-controllers.

**Conclusions:**

Host restriction factor expression typically scales with cellular activation levels. However, the elevated mRNA and protein expression of schlafen 11, despite low activation and viral load, violates the global pattern and may be a signature characteristic of HIV-1 elite control.

## Background

To date, there is no effective cure or prophylactic vaccine for HIV-1 infection. Antiretroviral therapy (ART) has dramatically decreased the morbidity and mortality associated with HIV-1 infection. However, there is a demand for alternative clinical management strategies due to the necessity of lifelong therapy, evolution of antiretroviral resistance, toxicity issues, and substantial costs of current regimens [1]. HIV-1 "elite controllers" naturally suppress HIV-1 to undetectable levels in the absence of ART, and therefore represent a promising model for a functional cure. The immunological determinants of control in these individuals may serve as foundations for novel antiretroviral strategies.

HIV-1 elite controllers appear to be a heterogeneous group, and the observed suppression of HIV-1 in these individuals has been attributed to a number of virologic, immunologic and genetic characteristics [2,3]. Although there are indications that viruses in elite controllers may be less virulent than strains in non-controllers [4], HIV-1 isolates from controllers often exhibit typical replication kinetics *in vitro* suggesting that reduced viral fitness cannot fully explain this phenomenon [5]. Multiple studies suggest that HIV-specific CD4+ [6] and CD8+ T cells [7] play a key role, and exhibit high functionality and proliferative capacity in elite controllers. Elite controllers have higher CD8+ T cell activation levels than ART-suppressed individuals, despite maintaining clinically undetectable levels of viral replication and lower CD4+ T cell activation levels [8]. Certain human leukocyte antigen (HLA) class I alleles such as HLA-B*57 and HLA-B*27 are overrepresented in elite controllers, and the protective effects of these alleles are thought to be CD8+ cell-mediated. However, not all elite controllers have protective HLA alleles [9], and some individuals with established protective alleles progress to disease rapidly [10]. CD8+ T cell responses alone cannot explain the elite controller phenotype, and other immunologic and molecular mechanisms likely play a role [9].

In addition to adaptive immune responses against HIV-1, cell-intrinsic mechanisms may play an important role in mediating resistance to HIV-1 infection in elite controllers. Genome wide mRNA expression studies suggest that a transcriptional profile signature of CD4+ T cells is associated with HIV-1 elite control and viral set point in viremic individuals [11,12]. In support of target cell-associated signature characteristics, CD4+ T cells from elite controllers may exhibit decreased susceptibility to HIV-1 infection *ex vivo* as compared to cells from viremic individuals, and cellular susceptibility to HIV-1 in controllers is predictive of reservoir size (total cell-associated HIV-1 DNA levels) [13,14]. However, this observation is controversial, and other studies report conflicting results [15-17]. Cell-intrinsic factors that contribute to HIV-1 control may include a number of recently identified proteins that restrict HIV-1 replication in target cells, and provide the host with a pre-mobilized defense against retroviral infection. The most widely recognized restriction factors are TRIM5α [18], APOBEC3G [19], and BST2/tetherin [20,21], and a number of additional factors with anti-HIV-1 activity have been identified and characterized in recent years. Our group recently published data suggesting that the BST-2/tetherin restriction factor plays a critical role in the interferon-mediated suppression of HIV-1 viremia in chronically infected individuals [22]. Although a few reports have examined the relevance of single factors (e.g. APOBEC3G) to HIV-1 plasma viral load and elite control [23], the overall contribution of host restriction mechanisms to HIV-1 elite control remains to be elucidated [2].

To address the hypothesis that cellular restriction of HIV-1 replication plays a significant role in the observed suppression of HIV-1 in elite controllers, we comprehensively compared restriction factor expression patterns and cellular activation levels in CD4+ T cells and T cell subsets between elite controllers, HIV-1-infected (untreated) non-controllers, ART-suppressed, and uninfected individuals enrolled in the UCSF SCOPE cohort. Restriction mechanisms suppress HIV-1 replication, while target cell activation promotes HIV-1 transactivation, replication, and production [24,25]; therefore, consideration of these two parameters in a synchronous fashion will allow us to gauge overall cell-intrinsic susceptibility to HIV-1 infection. We designed and implemented a custom TaqMan Low Density Array (TLDA) to measure the expression of 34 anti-HIV-1 restriction genes. The precise prerequisites for achieving the designation of host restriction factor are somewhat controversial. Moreover, this repertoire is by definition a moving target as new restriction mechanisms come to light. We relied on the following two minimal criteria for inclusion in our "**Cu**mulative **Re**striction" or "CuRe" TLDA: 1) Peer-reviewed, published evidence of direct inhibition of HIV-1 replication *in vitro*, and 2) Detectable expression in human peripheral blood mononuclear cells. In addition to bona fide, extensively characterized restriction factors such as APOBEC3G and BST-2/tetherin, the CuRe array measures a number of recently identified candidate anti-HIV-1 restriction factors to maximize the breadth, impact and generalizability of our translational study. Some of these factors may play functional roles outside of antiretroviral defense, and as of yet, co-evolutionary studies revealing canonical signatures indicative of historical host-pathogen conflict have not been performed across all sampled gene targets. However, all factors in the CuRe array meet the essential, minimal definition of a host restriction factor, and function in a cell-autonomous manner to suppress HIV-1 replication.

In addition to considering the individual expression of 34 anti-HIV-1 restriction genes, we created an intuitive mathematical construct to represent the overall, cumulative anti-HIV-1 restriction capacity associated with each sample. This metric, or "CuRe score", captures the cumulative fold-difference in antiviral gene expression with respect to a control individual.

## Results

### Schlafen 11 expression is elevated in elite controllers

We implemented the "CuRe" (Cumulative Restriction) TLDA to measure the mRNA expression of 34 anti-HIV-1 genes in CD4+ T cells from 48 subjects equally representing four HIV-1 disease states (12 elite controllers, 12 non-controllers, 12 ART-suppressed, and 12 HIV-1-uninfected individuals). The complete list of surveyed genes along with their respective reported anti-HIV-1 roles is presented in Table 1, precise disease state definitions are described in Table 2, and individual subject characteristics are documented in Additional file 1: Table S1. To represent overall HIV-1 inhibitory potential, we defined the CuRe score as the cumulative fold-difference in restriction factor expression with respect to a control individual (described in greater detail in the Methods section).

**Table 1:** List of the 34 anti-HIV-1 host restriction factors measured by our CuRe array

**Name**	**NCBI ENTREZ gene description**	**Key anti-HIV-1 role(S)**	**REFS**
APOBEC3 (A-H)	Apolipoprotein B mRNA editing enzyme, catalytic polypeptide-like3	Hypermutation; lethal mutations in viral DNA; Inhibition of reverse transcription; Inhibition of integration	[19,26-33]
TRIM family (11 members)	Tripartite motif family	Targeting of viral capsid; Inhibition of viral transcription	[18,34-36]
BST2/tetherin	Bone marrow stromal cell antigen 2	Blocks release of enveloped viruses	[20,21]
CDKN1A (P21)	Cyclin-dependent kinase inhibitor 1A (p21, Cip1)	Blocks reverse transcription; Blocks RNA transcription by reducing activity of CDK9	[13,37,38]
PAF1	Paf1, RNA polymerase II associated factor	Inhibits early events of viral life cycle from reverse transcription to integration	[39]
CTR9	Ctr9, Paf1/RNA polymerase II complex component	Inhibits early events of viral life cycle from reverse transcription to integration	[39]
RTF1	Rtf1, Paf1/RNA polymerase II complex component	Inhibits early events of viral life cycle from reverse transcription to integration	[39]
EIF2AK2 (PKR)	Eukaryotic translation initiation factor 2-alpha kinase 2	Inhibits viral protein translation by protein phosphorylation; promotes innate immune signaling	[40]
HERC5	HECT domain and RLD 5	Blocks early stage of retroviral particle assembly	[41]
IFITM Family (3 members)	Interferon induced transmembrane protein	Inhibits cytosolic entry	[42]
ISG15	ISG15 ubiquitin-like modifier	Blocks interaction between HIV-1 Gag and Tsg101 (ESCRT-I) required for efficient budding of HIV-1	[43]
MOV10	Mov10, Moloney leukemia virus 10, homolog	Inhibits proteolytic processing of Gag and reverse transcription	[44]
RNASEL	Ribonuclease L (2',5'-oligoisoadenylate synthetase-dependent)	Cleaves single-stranded RNA in U-rich sequences; activates antiviral innate immunity	[45]
RSAD2 (Viperin)	Radical S-adenosyl methionine domain containing 2	Inhibits viral production	[46]
SAMHD1	SAM domain and HD domain 1	Inhibits HIV replication in myeloid cells, probably by regulating cellular dNTP supply	[47]
SLFN11	Schlafen family member 11	Inhibits viral protein synthesis	[48]

**Table 2:** Definitions of HIV-1 disease states

**Patient category**	**Definition**
Elite Controllers	Antiretroviral therapy-naïve subjects who have at least one year duration of documented viral loads that are below the level of detection of conventional assays up until the date of specimen acquisition.
Non-controllers	Subjects who have had at least one documented viral load above 10,000 copies/ml who are not taking antiretroviral therapy.
ART Suppressed	Subjects taking antiretroviral therapy who have had at least three months duration of documented viral loads that are below the level of detection on conventional assays.

**Figure 1:**
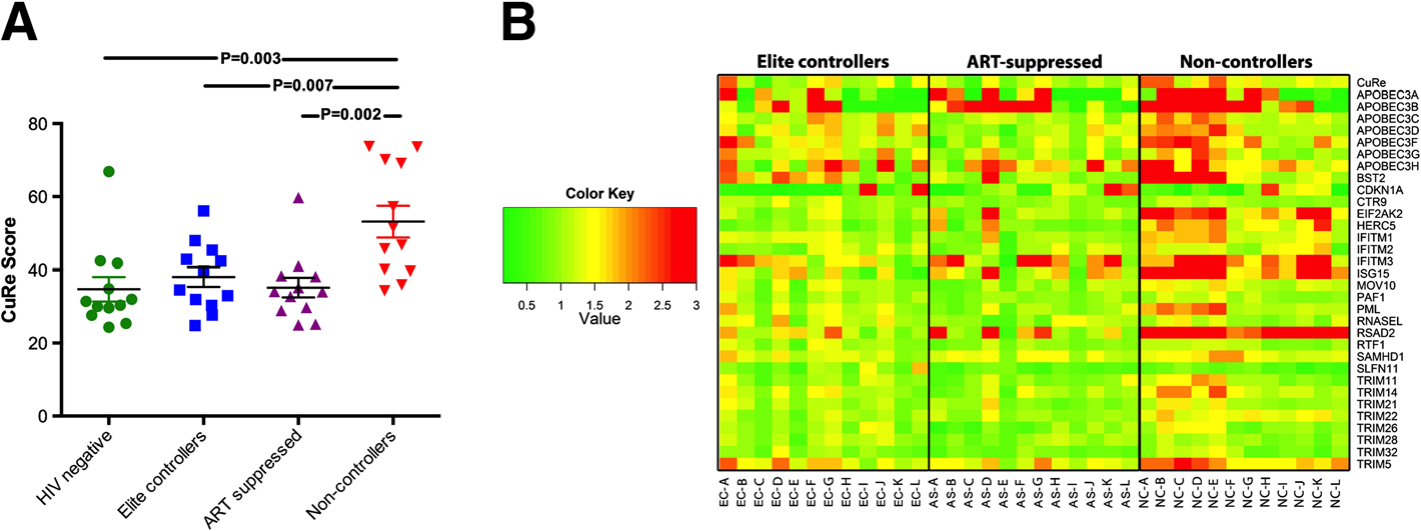
**Relationship between restriction factor expression and HIV-1 disease state. (A)** CuRe (**Cu**mulative **Re**striction) scores across HIV-1 disease states. Reported p-values were obtained using unpaired t tests. **(B)** Heat map representing expression of individual restriction genes across HIV-1 disease states. Fold-differences of each gene’s expression level in relation to the median values for each gene in the HIV-negative control group are reported. Yellow coloring indicates value of 1 (expression values equivalent to the median of HIV-negative controls). Red coloring indicates elevated relative expression, and green coloring indicates suppressed relative expression. Each column represents a single individual.

CD4+ T cells are the primary HIV-1 target cells within peripheral tissues, and therefore gene expression in this cellular subset is likely to be most relevant to viral production and propagation. CD4+ T cells were negatively-selected from freshly-collected blood with near 100% purity as described previously [22]. Expression of anti-HIV-1 genes in CD4+ T cells, as summarized by the CuRe score, was significantly higher in non-controllers as compared to elite controllers (p = 0.007), ART-suppressed (p = 0.002), or uninfected controls (p = 0.003) (Figure 1). A complete list of relative copy numbers for each restriction factor, and p-values for all inter-disease state gene expression comparisons are presented in Table 3 and Additional file 1: Table S2, respectively. These data suggest that overall, global restriction factor expression is not enhanced in elite controllers with respect to other HIV-1 disease groups, in contrast to the near-global induction of antiviral restriction mechanisms observed in individuals undergoing interferon-α therapy (unpublished observations, M. Abdel-Mohsen *et al.*).

**Table 3:** **Anti-HIV-1 restriction factor mRNA relative copy number**^**1**^

	**HIV negative controls**	**Elite controllers**	**ART-suppressed**	**Non-controllers**
**APOBEC3A**	9.45 ± 2.3	7.95 ± 3.2	7.25 ± 1.6	10.23 ± 2.9
**APOBEC3B**	2.76 ± 0.5	3.230.8	4.91 ± 1.2	11.65 ± 2.7
**APOBEC3C**	419.62 ± 42.3	509.99 ± 38.9	393.1 ± 27.1	545.81 ± 49.5
**APOBEC3D**	96.42 ± 12.4	125.44 ± 11.2	92.44 ± 8.7	139.17 ± 14
**APOBEC3F**	2 ± 0.3	2.73 ± 0.4	1.88 ± 0.2	3.35 ± 0.4
**APOBEC3G**	66.29 ± 9	87.22 ± 7.4	67.16 ± 6.6	84.02 ± 9.7
**APOBEC3H**	10.88 ± 2.2	12.89 ± 2.6	12.53 ± 1.6	16.74 ± 3
**BST2**	59.15 ± 9.1	81.19 ± 9.9	62.31 ± 8.5	108.6 ± 16.5
**CDKN1A (P21)**	90.75 ± 25.8	74.43 ± 41.1	77.39 ± 29.9	48.6 ± 11.5
**CTR9**	170.92 ± 11	175.81 ± 11.1	146.7 ± 5.6	164.3 ± 8.1
**EIF2AK2**	416.56 ± 11.8	359.92 ± 14.5	434.52 ± 11.5	805.28 ± 8.1
**HERC5**	179.53 ± 28.7	129.72 ± 9.1	146.37 ± 22.2	250.33 ± 20.9
**IFITM1**	12102.96 ± 624.2	13852.2 ± 778	11789.6 ± 1190	18163.4 ± 1222.4
**IFITM2**	2325.19 ± 161.3	2442.24 ± 178	2256.65 ± 195.9	2903.74 ± 239.8
**IFITM3**	66.43 ± 17.1	85.81 ± 13.6	97.44 ± 16	143.82 ± 20.9
**ISG15**	17.85 ± 3.7	16.82 ± 2.3	26.91 ± 8.3	56.58 ± 9.1
**MOV10**	151.25 ± 10.6	162.57 ± 14.7	137.56 ± 10.6	190.16 ± 9.9
**PAF1**	95.25 ± 3.6	98.88 ± 6.4	87.55 ± 4.2	102.38 ± 6.4
**RNASEL**	34.34 ± 2.9	37.96 ± 3.5	31.75 ± 2.9	34.32 ± 2.5
**RSAD2 (Viperin)**	29.86 ± 12.8	23.2 ± 2.5	37.11 ± 11.2	102.81 ± 15.4
**RTF1**	322.64 ± 18.2	323.08 ± 24.1	276.97 ± 16.5	322.93 ± 15.7
**SAMHD1**	1322.37 ± 159.4	1420.63 ± 95.8	1470.53 ± 78	1685.26 ± 92.4
**SLFN11**	92.4 ± 10.1	80.98 ± 10.3	54.64 ± 3.4	57.44 ± 4.6
**TRIM11**	85.09 ± 7.4	82.25 ± 6.3	72.81 ± 6.4	97.63 ± 10.1
**TRIM14**	296.58 ± 25.6	351.14 ± 26.8	302.04 ± 24	460.72 ± 42.5
**TRIM19 (PML)**	180.3 ± 14.5	203.55 ± 16.1	169.58 ± 13	267.52 ± 27.6
**TRIM21**	128.44 ± 8.9	133.74 ± 8.6	120.86 ± 10.3	148.63 ± 11.1
**TRIM22**	1547.88 ± 124.4	1457.21 ± 93.4	1418.98 ± 78.9	2049.2 ± 89.4
**TRIM26**	197.14 ± 16.2	188.7 ± 13.3	146.16 ± 14	201.34 ± 18.4
**TRIM28**	545.06 ± 27	562.97 ± 36.6	499.71 ± 32.2	527.79 ± 28.5
**TRIM32**	178.17 ± 12.7	173.69 ± 14	139.87 ± 7.3	180.89 ± 16.9
**TRIM5**	68.18 ± 10.6	86.44 ± 7.2	77.97 ± 5.7	108.67 ± 6.8

^1^Mean ± standard error.

**Figure 2:**
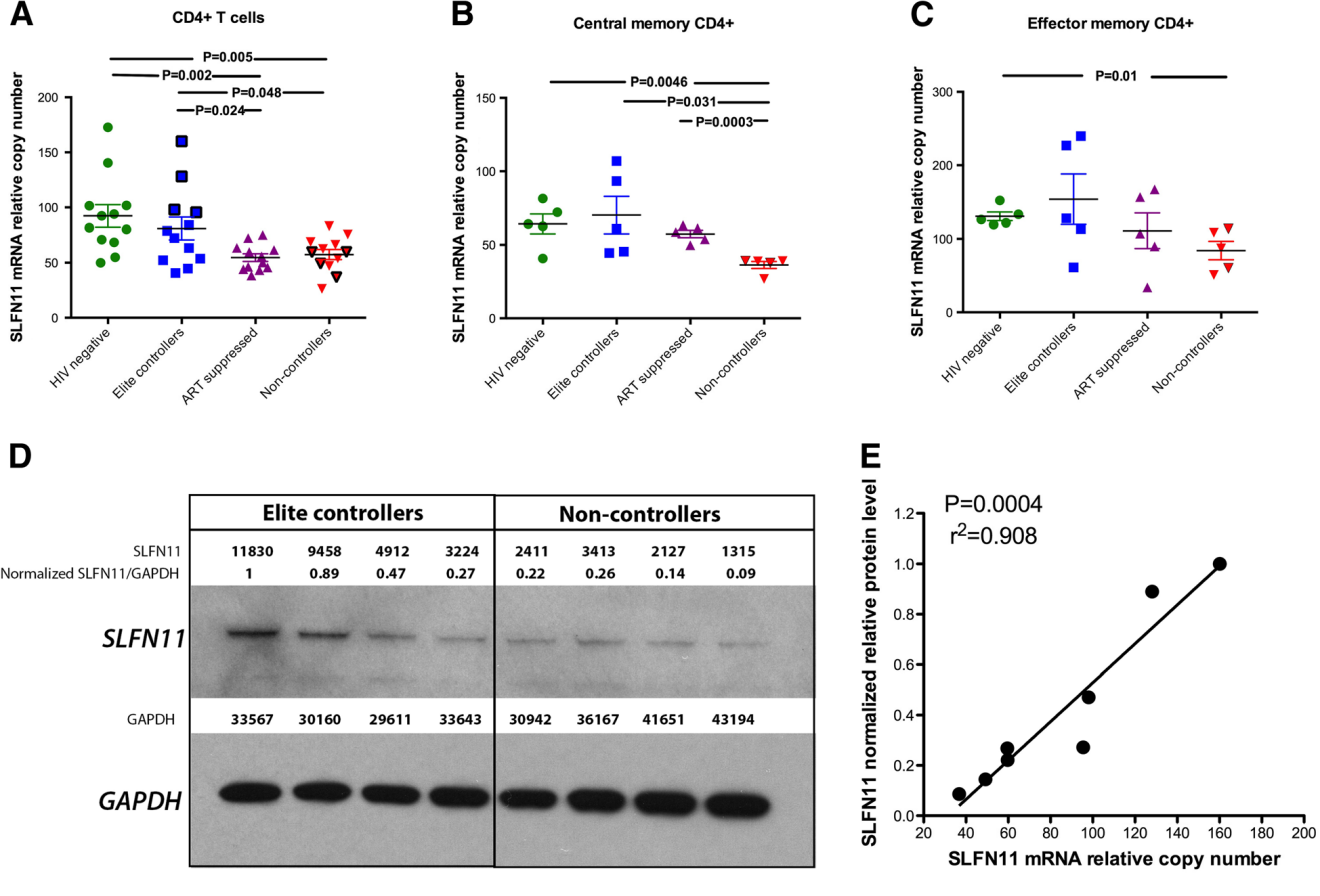
**Elevated expression of schlafen 11 in HIV-1 elite controllers. (A)** schlafen 11 (SLFN11) expression in unfractionated CD4+ T cells across disease states. Data points involved in subsequent protein characterization are highlighted with black bordering. **(B)** SLFN11 expression in central memory CD4+ T cells. **(C)** SLFN11 expression in effector memory CD4+ T cells. Reported p-values in panels **A-C** were obtained using unpaired t tests. **(D)** SLFN11 protein expression in elite controllers and non-controllers, as determined by western blot. Immunoblotting bands were quantified with ImageJ64 software. The quantified SLFN11 protein expression levels were normalized to corresponding GAPDH protein levels to ensure equal loading. **(E)** Correlation between SLFN11 normalized protein level and mRNA relative copy number. A Spearman’s rank test was used to evaluate the significance of the correlation.

Although CuRe scores and the majority of individual gene-specific mRNA measurements were highest in viremic non-controllers with respect to other groups represented in our study (Figure 1), we sought to determine if particular anti-HIV-1 restriction factors violated this average behavior and exhibited high mRNA expression levels in HIV-1 elite controllers. We performed iterative univariate analyses to identify genes that were maximally expressed in elite controllers with respect to other HIV-1-infected individuals. Our analyses revealed that a single factor, schlafen 11, was expressed at a significantly higher level in CD4+ T cells from elite controllers with respect to viremic, untreated non-controllers (p = 0.048) and ART-suppressed (p = 0.024) individuals (Figure 2A). Schlafen 11 is a recently identified anti-HIV-1 restriction factor that potently suppresses HIV-1 replication by codon usage-based inhibition of HIV-1 protein synthesis [48].

We next performed an exploratory analysis of the mRNA expression of schlafen 11 in central memory and effector memory CD4+ T cell subsets. Multiple reports suggest that elite controllers and long-term non-progressors harbor higher relative numbers of central memory CD4+ T cells, and these cells exhibit distinct phenotypic properties from viremic non-controllers [49,50]. Flow-based sorting was used to isolate central and effector memory populations in a subset of 20 individuals (5 of each disease state), and the CuRe array was implemented to measure restriction gene expression. Our data from these cellular subsets reveal that central memory CD4+ T cells from elite controllers (Figure 2B), but not effector memory cells (Figure 2C), express significantly higher levels of schlafen 11 (p = 0.031) than corresponding cellular subsets from viremic non-controllers (although a trend was apparent in effector memory cells).

To confirm that our schlafen 11 mRNA expression data were recapitulated at the protein level, we measured schlafen 11 protein expression in CD4+ T cells from a subset of four elite controllers and four non-controllers, chosen based on range of mRNA expression and availability of remaining specimens (data points in Figure 2A corresponding to samples chosen for subsequent protein characterization are highlighted with black bordering). Our western blots demonstrate that schlafen 11 protein expression (normalized to GAPDH protein level to control for loading) is elevated in elite controllers with respect to non-controllers, validating our mRNA-based observations (Figure 2D). Moreover, schlafen 11 normalized protein levels and mRNA relative copy numbers exhibit a near-perfect correlation, suggesting that mRNA quantitation is a reliable strategy for measuring schlafen 11 expression in primary cells (Figure 2E).

### Restriction factor expression *in vivo* is correlated with CD4+ T cell activation

**Figure 3:**
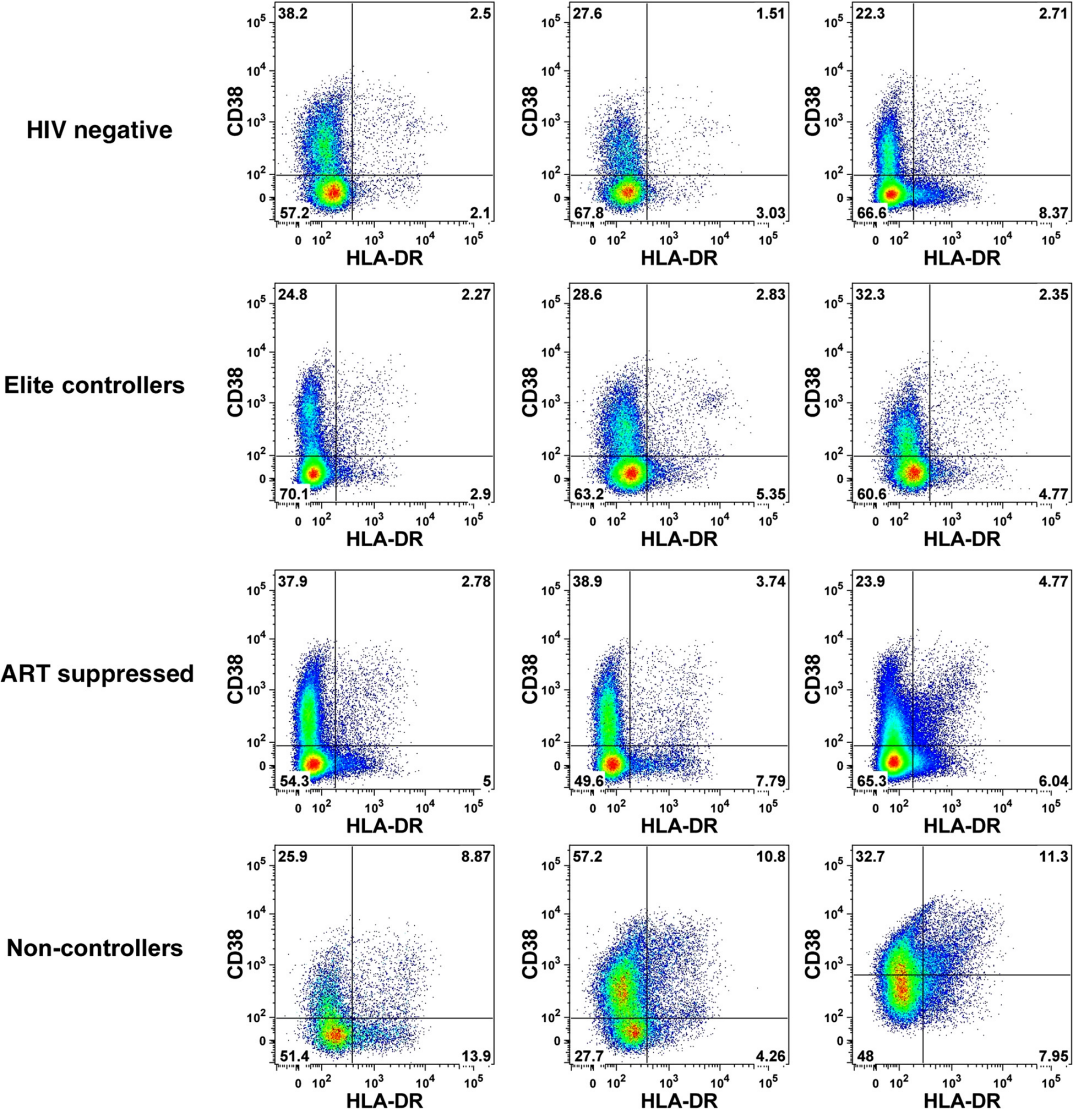
**Frequency of activated CD4+ T cells across HIV-1 disease states.** CD38 and HLA-DR expression on the surface of CD4+ T cells was measured by flow cytometry. Plots for three representative, median individuals are included for each HIV-1 disease state. Percentages of activated cells (co-expressing CD38 and HLA-DR) are reported in the upper-right quadrant of each plot.

**Figure 4:**
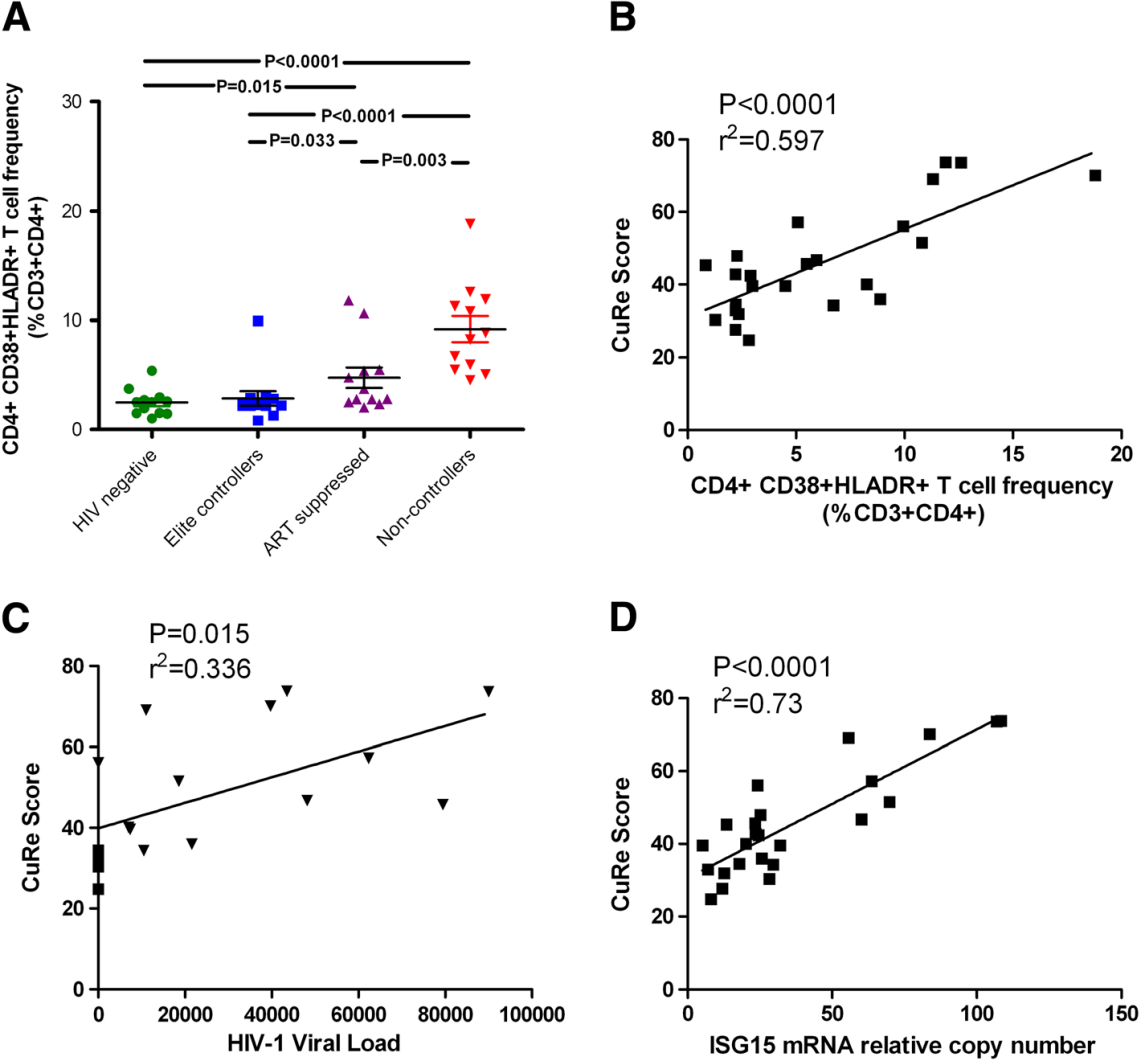
**Immunologic and virologic correlates of host restriction factor expression. (A)** Frequency of activated (CD38+ HLA-DR+) CD4+ T cells across disease states. Reported p-values were obtained using unpaired t tests. Correlations between CuRe score and CD4+ T cell activation, HIV-1 viral load, and ISG15 expression in HIV-1-infected, untreated individuals (elite controllers and non-controllers) are reported in **(B)**, **(C)**, and **(D)**, respectively. Correlations were evaluated using Pearson’s r tests.

Based on our observation that restriction factor expression was highest in non-controllers, we hypothesized that restriction gene expression fluctuates in tandem with CD4+ T cell activation levels. We used flow cytometry to determine the relative levels of CD4+ T cell activation in all study subjects. T cell activation levels were reported as a percentage of CD4+ T cells co-expressing HLA-DR and CD38, as well as the mean fluorescence intensity (MFI) of HLA-DR. In our study population, non-controllers had a higher frequency of activated CD4+ T cells compared to elite controllers (p < 0.0001), ART-suppressed (p = 0.003), or uninfected individuals (p < 0.0001) (Figure 3, Figure 4A). We additionally observed a higher frequency of activated CD4+ T cells in the ART-suppressed group compared to elite controllers (p = 0.033) and uninfected individuals (p < 0.015) (Figure 4A). The CD4+ activation profiles reported here recapitulate previously reported data [8]. Focusing on all HIV-1 infected, untreated individuals, we identified statistically significant correlations between the CuRe score and frequency of activated CD4+ T cells (r^2^ = 0.597, p < 0.0001) (Figure 4B), and HIV-1 plasma viral load (r^2^ = 0.336, p = 0.015) (Figure 4C). We also examined the relationship between the CuRe score and the expression of interferon-stimulated gene 15 (ISG15), a marker of interferon exposure, and observed a strong correlation (r^2^ = 0.73, p < 0.0001) (Figure 4D).

### Elite controllers exhibit a distinct restriction factor expression profile from ART-suppressed individuals

**Figure 5:**
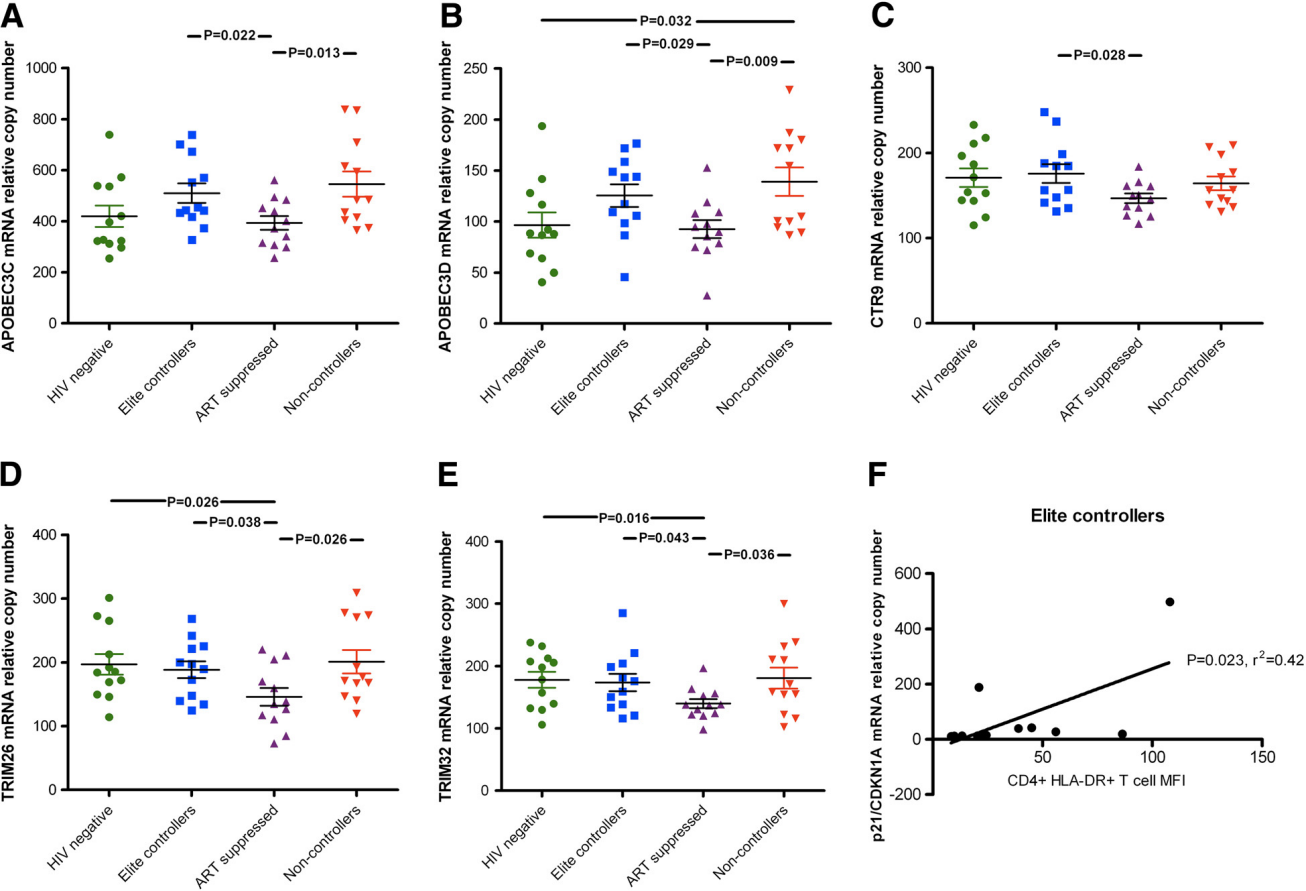
**Elevated expression of restriction factor genes in HIV-1 elite controllers with respect to ART-suppressed individuals.** Five restriction factors were significantly elevated in elite controllers with respect to ART-suppressed subjects: **(A)** APOBEC3C, **(B)** APOBEC3D, **(C)** CTR9, **(D)** TRIM26, and **(E)** TRIM32. Gene expression between groups was compared using unpaired t tests. **(F)** The expression of CDKN1A/p21 was positively correlated with CD4+ T cell activation in elite controllers. There were no correlations between gene expression and activation in ART-suppressed individuals. Correlations were evaluated using Pearson’s r tests.

As HIV-1 replication apparently drives expression of many restriction factors, we compared gene expression in the two "aviremic" infected groups (controllers and ART-suppressed). Our rationale was that differences in gene expression between these groups would be minimally confounded by differences in HIV-1 antigen levels, and may provide insights into factors which are mechanistically associated with virus control. In this two-way comparison, five anti-HIV-1 restriction genes were significantly elevated in elite controllers when compared to ART-suppressed subjects: APOBEC3C (p = 0.022), APOBEC3D (p = 0.029), CTR9 (p = 0.028), TRIM26 (p = 0.038), and TRIM32 (p = 0.044) (Figure 5A-E). No genes were elevated in ART-suppressed individuals with respect to controllers. The enhanced expression of these five genes in elite controllers violates the typical positive correlation with CD4+ T cell activation, as does schlafen 11. We examined the correlations between the expression of restriction genes and CD4+ T cell activation within the two aviremic, HIV-1-infected groups. CDKN1A/p21 was the only gene that exhibited a significant correlation in elite controllers; its expression was positively correlated with activation in controllers (r^2^ = 0.42, p < 0.023) (Figure 5F), while there were no significant correlations between gene expression and activation in ART-suppressed individuals.

## Discussion

Our overall objective was to determine the relevance of host-encoded anti-HIV-1 restriction factors to HIV-1 elite controller status. We measured the relative expression levels of 34 different antiviral factors in CD4+ T cells from elite controllers to ascertain if one or more restriction genes are associated with the control of HIV-1 *in vivo*. Overall restriction factor expression (as measured by the CuRe score) exhibited significant, pronounced relationships with T cell activation and ISG15 expression (an indicator of interferon levels), and a less pronounced positive correlation with viral load. This pattern likely reflects a scenario in which restriction factor expression is primarily driven by cellular activation and interferon exposure *in vivo,* mirroring recent data from our group derived from *in vitro* experiments [51] and studies of HLA-B*57-positive healthy donors [52]. The moderate correlation with viral load likely reflects an indirect association (since activation and interferon expression are driven by viral antigen). This pattern parallels data describing positive correlations between the breadth and magnitude of anti-HIV-1 CD8+ responses and viral load observed in structured treatment interruption studies [53]. Natural variation in restriction factor mRNA expression on a global level, considered independently of other cellular and immunologic parameters, does not appear to be a prognostic indicator of effective viral control *in vivo*. However, overall restriction factor expression may serve as a prognostic indicator of HIV-1 suppression within the context of exogenous interferon-α treatment, when expression and activity of several factors is induced to supraphysiologic levels (unpublished observations, M. Abdel-Mohsen *et al.*).

Schlafen 11 [48] was the only gene in our array that exhibited significantly higher expression in elite controllers as compared to both viremic non-controllers and ART-suppressed groups. This suggests that schlafen 11 may play a role in the suppression of HIV-1 *in vivo*, by selectively inhibiting the synthesis of HIV-1 proteins via tRNA limitation and codon-based discrimination (HIV-1 favors A/T-rich codons in reflection of its skewed base composition). Moreover, expression data from our exploratory analyses of sorted T cell subsets indicate that schlafen 11 may specifically contribute to the distinct phenotypic signature and resilience associated with CD4+ central memory cells from HIV-1 elite controllers [49,50]. However, despite the fact that we were able to identify statistically significant differences in expression levels between patient groups in these subsets that recapitulated the hierarchy observed in unsorted CD4+ cells, these observations warrant validation using larger sample sizes.

The hierarchy of schlafen 11 gene expression violates the typical linkage with cellular activation levels and interferon exposure. In addition, the lack of a significant difference between uninfected individuals and elite controllers in juxtaposition with significantly reduced expression levels in viremic non-controllers and ART-suppressed HIV-1-infected individuals suggests that controllers may be protected from viral downregulation of the schlafen 11 factor. This pattern may be driven by host and/or viral determinants; elite controllers may harbor a genetic variant of the schlafen 11 protein that is less susceptible to HIV-1 evasion and modulation. Alternatively, elite controllers may be infected by genetic variants of HIV-1 that exhibit attenuated capacity to downregulate schlafen 11, mirroring reported observations that HIV-1 strains in elite controllers express weak or defective versions of the Nef and Vif proteins [4]. However, as of yet, this is speculative, since a schlafen 11 antagonistic factor in HIV-1 remains to be identified. Another possibility stems from the characteristics of uninfected controls included in our study; this population, reflecting the composition of uninfected individuals enrolled in the SCOPE cohort, is enriched for highly HIV-exposed seronegative individuals, and factors such as schlafen 11 that contribute to viral control may also protect against HIV-1 acquisition. This is supported by the observation that amongst uninfected controls in our study, highly-exposed individuals express schlafen 11 at significantly higher levels than individuals with minimal exposure (data not shown).

It is important to note that despite statistically significant differences between patient groups, not all elite controllers in our study expressed schlafen 11 at elevated levels. Some controllers exhibit high schlafen 11 mRNA and protein expression, while other controllers are indistinguishable from non-controllers. This pattern mirrors other immunologic measurements of elite controllers; these individuals exhibit highly heterogeneous neutralizing antibody responses and T cell phenotypes [2,3,54]. This variability may reflect a scenario whereby some elite controllers are immunologically adapted to retroviral infection, while others simply maintain undetectable viremia due to poor replicative fitness of the infecting viral strain.

Five restriction factors (APOBEC3C, APOBEC3D, TRIM26, TRIM32, and CTR9) were elevated in elites when compared specifically to ART-suppressed individuals, who have similar levels of residual viremia. The elevated expression of these five genes, including APOBEC3 cytidine deaminases and TRIM-family members, constitutes an elite controller-specific signature. Moreover, the expression of CDKN1A/p21 scales with CD4+ T cell activation in elites (while no such correlations were observed in treated patients). CDKN1A/p21, a potent inhibitor of cyclin-dependent kinases, has recently been proposed to exert *in vivo* anti-HIV-1 activity, and is believed to be overexpressed in CD4+ T cells from elite controllers [13]. However, other groups report that CDKN1A/p21 is not directly involved in HIV-1 restriction [14]. We found no statistically significant difference in the expression levels of CDKN1A/p21 in elite controllers compared to any of the other three disease states. This observation remained consistent when the mRNA expression of these factors was examined specifically in central memory and effector memory CD4+ T cell subsets. The observation that expression of p21 was significantly correlated with CD4+ T cell activation in controllers, but not in any of the other groups included in our study, is provocative. This suggests that the inducibility and responsiveness of this defense factor to viral antigen, rather than constitutive overexpression, may contribute to the elite controller phenotype.

There are certain caveats associated with these data and their interpretation. Unlike studies involving *in vitro* or animal models, it is difficult to unequivocally demonstrate a causal relationship between restriction factor expression and the control of HIV-1 using a patient-based study design. Consideration of our findings within the context of the robust literature describing a causal relationship between these restriction factors and HIV-1 suppression *in vitro* (Table 1) lends credibility to these *in vivo* observations. Another caveat involves our characterization of mRNA expression levels. Although concordance between mRNA and protein expression has been demonstrated for several of our studied factors, mRNA may not always accurately predict protein concentrations. Although we performed confirmatory protein quantitation to validate elevated schlafen 11 expression in elite controllers, cellular protein concentrations may not always accurately reflect antiviral activity *in vivo*. Functional assays using clinical specimens from elite controllers will complement and extend these findings.

## Conclusions

Our data suggest that overall restriction factor gene expression, captured in our CuRe score, is highest amongst non-controllers, likely a reflection of increased viral antigen and interferon exposure. However, the codon-usage-based inhibitor of HIV protein synthesis schlafen 11 is a notable exception to this pattern. Elevated schlafen 11 expression is a signature characteristic of HIV-1 elite control and may play a role in the suppression of HIV-1 *in vivo*. These findings complement earlier reports suggesting that CD4+ T cells from controllers are refractory to HIV-1 infection *ex vivo*. Continued investigation into the regulation, tissue-specific expression, and antiretroviral capacity of host restriction factors will likely benefit the development of therapeutic and curative strategies for HIV-1 infection.

## Methods

### Subjects and specimen processing

60 cc of blood was collected prospectively from 48 individuals enrolled in the UCSF SCOPE Cohort. The research was approved by the relevant institutional review boards, and all human participants gave written informed consent. Disease state definitions are described in Table 2, and individual subject characteristics are documented in detail in Additional file 1: Table S1. Plasma RNA viral load and CD4+ T cell counts were measured at all patient visits. Total blood from all subjects was processed with Ficoll-Plaque PLUS and 10^6^ PBMCs were immediately subject to CD4+ cell enrichment. CD4+ T cells were enriched from fresh PBMCs using the EasySep Human CD4+ T Cell enrichment magnetic kit (StemCell Technologies), according to the manufacturer’s instructions. Total RNA extraction was performed directly after enrichment.

### Cell sorting

A subset of 20 individuals (5 of each disease status) were chosen for sorting; four CD4+ T cell subpopulations (naïve, central memory, transitional memory, and effector memory cells) were isolated using a FACS-based sorting procedure. PBMCs were isolated by Ficoll density gradient, stained with aqua amine reactive dye, and then stained with fluorescently conjugated monoclonal antibodies against CD3, CD4, CD45RO, CD27, CCR7, CD57 and CD14. After gaining on lymphocytes and singlets, and excluding non-viable cells (using the amine reactive dye) and CD14+ monocytes, CD3+CD4+ cells were sorted as follows: [1] Naïve (CD45RO-CCR7+CD27+CD57-); [2] Central Memory (CD45RO+CCR7+CD27+); [3] Transitional Memory (CD45RO+CCR7-CD27+); and [4] Effector Memory (CD45RO+CCR7-CD27-) (Additional file 1: Figure S1).

### Gene expression profiling

Total RNA was extracted from enriched CD4+ T cells using 700 μl of Qiazol reagent (Qiagen), followed by 15 min centrifugation at 12,000 g at 4°C. RNA was extracted from the aqueous layer using the miRNeasy Mini kit (Qiagen) with on-column DNAase treatment (Qiagen RNase-Free DNase Set) and eluted in 30 μl of RNase-free water. DNase-treated, clean RNA was transcribed into cDNA using random primers and the SuperScript® VILO™ cDNA Synthesis Kit (Invitrogen), according to manufacturer’s instructions. Quantitative real-time PCR utilized custom-made TaqMan® Low Density Arrays (TLDA) from Applied Biosystems following the manufacturer’s instructions. TaqMan Low Density Array (TLDA) cards (Applied Biosystems, Foster City, CA) are 384-well microfluidic cards with eight ports, each containing 48 connected wells. The primers and probes for each assay were preloaded and dried onto the designated duplicate wells. All probes used on our card are conjugated to 6-carboxyfluorescein (FAM). Black hole quencher 1 (BHQ1) was used as a quencher for all probes. The Cumulative Restriction (CuRe) TLDA card is designed to run 4 samples in duplicate on one card. Each port tests against 34 different antiviral genes and a panel of housekeeping genes. All assays and their respective target genes are listed in Table 1. Thermal cycling was performed using an ABI ViiA™ 7 Real-Time PCR System. Up to 450 ng cDNA in 200 μl of Applied Biosystems TaqMan Universal PCR Master Mix with UNG was loaded onto the designated ports of the TLDA plates. Data was analyzed using ABI ViiA™ 7 software. A panel of 6 housekeeping genes was included in the TLDA plates (GAPDH, 18S, ACTB, PPIA, RPLP0, and UBC). RPLP0 was identified as the most stably expressed gene from those 6 housekeeping genes among all samples using the GeNorm algorithm [55]. Therefore, raw cycle threshold numbers of amplified gene products were normalized to the housekeeping gene, RPLP0, (60S acidic ribosomal protein P0) to control for cDNA input amounts. Fold induction was determined using the comparative Ct method [55].

### CuRe score calculation

The expression value for the i^th^ gene is notated as e_i_, i = 1,2,…,n, assuming there are n genes. Missing values were imputed using the minimum expression value across samples for each gene, respectively. The median gene expression profile is calculated for the HIV-1-uninfected control group. A reference sample with the maximum number of genes that are closest to the median gene expression profile is the chosen from the uninfected control group. The reference expression value for the i^th^ gene is notated as r_i_, i = 1,2,…,n. The gene expression profile for each sample was standardized by calculating the ratio against the reference sample. The CuRe score for a sample is the cumulative fold-difference in antiviral gene expression with respect to a control individual, expressed by the following formula:

CuRe=∑i=1nei/ri

### Western blotting

Eight cryopreserved PBMC samples (four elite controller, four non-controller) were chosen for protein quantitation. CD4+ T cells were negatively selected from PBMC as described previously. 15 μl of cellular lysates in NuPAGE LDS Sample Buffer with 2-mercaptoethanol (equal to 0.6 million cells) were resolved by NuPAGE 4-12% gel, then transferred to PVDF membrane. The membrane was blocked by 5% non-fat dry milk/TBST and incubated with anti-SLFN11 mAb (1:500 dilution, Santa Cruz Biotech SLFN11 Antibody (E-4): sc-374339) or anti-GAPDH (1:2500, Cell Signal Tech GAPDH (D16H11) XP® Rabbit mAb #5174) in 5% BSA/TBST at 4C overnight. The next day, the membrane was washed and incubated with Cell Signal Tech anti-mouse or anti-rabbit HRP antibodies respectively and developed with PerkinElmer Western Lightning ECL Pro. The immunoblotting bands were quantified with ImageJ64 software. The quantified SLFN11 protein expression levels were normalized to corresponding GAPDH protein levels to ensure equal loading.

### Flow cytometry

Cryopreserved PBMCs were quickly thawed, washed in FACS buffer (2 mM EDTA, 0.5% BSA in ice-cold PBS) and surface stained for 30 min at 4°C. Activation panel included the following antibodies: CD3-ECD (1:50, UCHT1, Beckman Coulter), CD4-PacificBlue (1:50, RPA-T4, Beckman Coulter), CD8-QDot605 (3B5, Invitrogen), CD14-APC/Cy7 (1:100, MΦP9, Becton Dickinson), CD19-APC/Cy7 (1:100, SJ25C1, BD Pharmingen), CD38-PE (1:50, HB7, BD Pharmingen), HLA-DR-FITC (1:200, L243 (G46-6), BD Pharmingen), and a live/dead marker (Invitrogen) emitting in the aqua wavelength. After staining, the cells were washed three times, fixed in 2% paraformaldehyde, and events were collected on a Becton Dickinson LSR-II, using FACS DIVA software (BD Biosciences). Data were analyzed using FlowJo software (version 9.3.2, TreeStar). FMOs were carried out for compensation purposes. T cell activation levels are reported as the percentage of CD4+ T cells co-expressing HLA-DR and CD38 and the mean fluorescence intensity (MFI) of HLA-DR.

### Statistical analysis

A battery of statistical tests including unpaired t tests and Pearson’s r correlation coefficient were applied to data using GraphPad Prism v5.0c.

## Abbreviations

APOBEC: Apolipoprotein B mRNA editing enzyme; BST-2: Bone marrow stromal cell antigen 2; SAMHD1: SAM domain and HD domain-containing protein 1; TRIM: Tripartite motif; ISG: Interferon-stimulated gene; CDKN1A: Cyclin-dependent kinase inhibitor 1A; PAF1: RNA polymerase II associated factor; EIF2AK2: Eukaryotic translation initiation factor 2-alpha kinase 2; HERC5: HECT domain and RLD 5; IFITM: Interferon induced transmembrane; MOV10: Moloney leukemia virus 10 homolog; SLFN11: Schlafen family member 11; TLDA: TaqMan® low density array; GAPDH: Glyceraldehyde 3-phosphate dehydrogenase; 18S: 18S ribosomal RNA; ACTB: Beta-actin; PPIA: Peptidylprolyl isomerase A; RPLP0: 60S acidic ribosomal protein P0; UBC: Ubiquitin C; HN: HIV negative controls; EC: Elite controllers; AS: ART suppressed; NC: Non-controllers.

## Competing interests

The authors declare that they have no competing interests.

## Authors’ contributions

MAM, RASR, TL, ES and SKP performed gene expression and flow cytometry-based experiments, ML and MD performed western blotting, and XD performed statistical analyses. RH and SGD coordinated patient recruitment. MAM, SKP, JKW, DFN, SGD, MSS and HEAG designed the studies. MAM and SKP wrote the paper. All authors read and approved the final manuscript.

## Supplementary Material

Additional file 1: Figure S1 FACS-based sorting of CD4+ T cell subpopulations. PBMCs were isolated by Ficoll density gradient, stained with aqua amine reactive dye, and then stained with fluorescently-conjugated monoclonal antibodies against CD3, CD4, CD45RO, CD27, CCR7, CD57 and CD14. After gaining on lymphocytes and singlets, and excluding non-viable cells (using amine reactive dye) and CD14+ monocytes, CD3+CD4+ cells were sorted as illustrated: (1) Naïve (CD45RO-CCR7+CD27+CD57-); (2) Central Memory (CD45RO+CCR7+CD27+); (3) Transitional Memory (CD45RO+CCR7-CD27+); and (4) Effector Memory (CD45RO+CCR7-CD27-). **Table S1.** Subject characteristics. **Table S2.** P values for inter-disease state gene expression comparisons. Click here for file
