# Supplementary material for: Expression profile of host restriction factors in HIV-1 elite controllers
Source: Retrovirology. 2013 Oct 16;10:106. doi: 10.1186/1742-4690-10-106 (PMC3827935; doi:10.1186/1742-4690-10-106)
Supplement: Additional file 1: Figure S1 — FACS-based sorting of CD4+ T cell subpopulations. PBMCs were isolated by Ficoll density gradient, stained with aqua amine reactive dye, and then stained with fluorescently-conjugated monoclonal antibodies against CD3, CD4, CD45RO, CD27, CCR7, CD57 and CD14. After gaining on lymphocytes and singlets, and excluding non-viable cells (using amine reactive dye) and CD14+ monocytes, CD3+CD4+ cells were sorted as illustrated: (1) Naïve (CD45RO-CCR7+CD27+CD57-); (2) Central Memory (CD45RO+CCR7+CD27+); (3) Transitional Memory (CD45RO+CCR7-CD27+); and (4) Effector Memory (CD45RO+CCR7-CD27-). Table S1. Subject characteristics. Table S2. P values for inter-disease state gene expression comparisons. [file 1742-4690-10-106-S1.docx]

Additional file Materials:


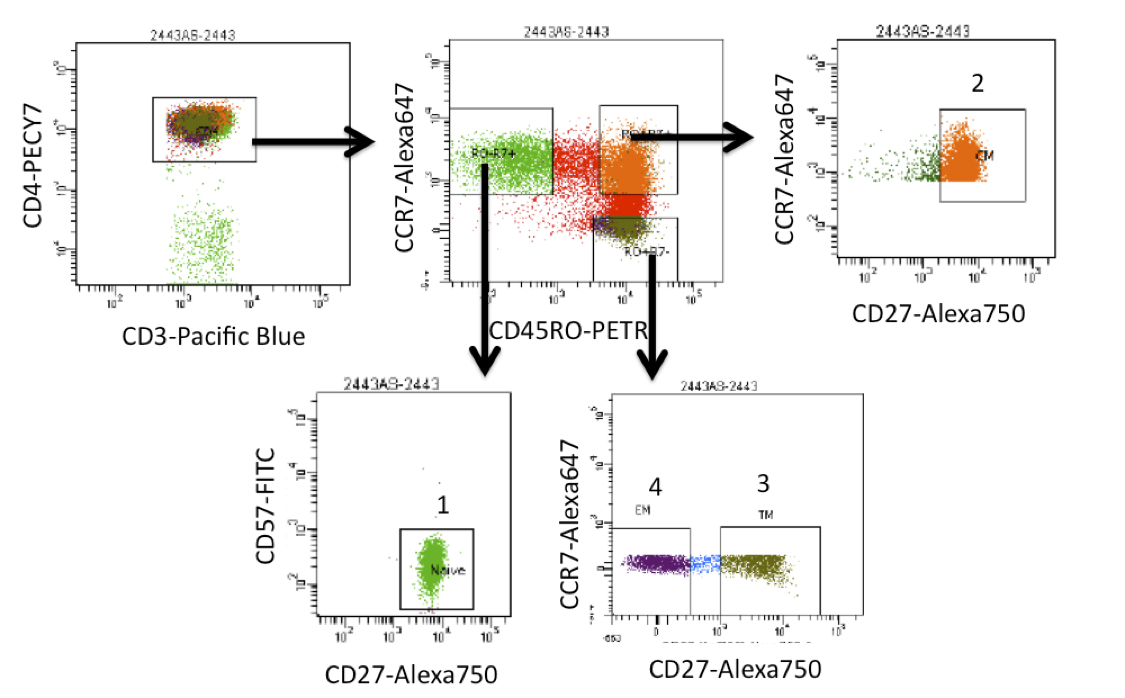


**Additional file Figure 1. FACS-based sorting of CD4+ T cell subpopulations.** PBMCs were isolated by Ficoll density gradient, stained with aqua amine reactive dye, and then stained with fluorescently-conjugated monoclonal antibodies against CD3, CD4, CD45RO, CD27, CCR7, CD57 and CD14. After gaining on lymphocytes and singlets, and excluding non-viable cells (using amine reactive dye) and CD14+ monocytes, CD3+CD4+ cells were sorted as illustrated: (1) Naïve (CD45RO-CCR7+CD27+CD57-); (2) Central Memory (CD45RO+CCR7+CD27+); (3) Transitional Memory (CD45RO+CCR7-CD27+); and (4) Effector Memory (CD45RO+CCR7-CD27-).

**Additional file Table 1.** Subject characteristics

|  | **Subject ID** | **Gender** | **Age (y)** | **CD4+ count (cells/μl)** | **CD4%** | **ART regimen** | **HIV-1 viral load (copies/ml)** |
| --- | --- | --- | --- | --- | --- | --- | --- |
| **HIV negative controls** | HN-A | M | 54 | 684 | 46 |  | Not Detected |
|  | HN-B | M | 49 | 1263 | 49 |  | Not Detected |
|  | HN-C | M | 45 | 460 | 40 |  | Not Detected |
|  | HN-D | M | 52 | 1077 | 52 |  | Not Detected |
|  | HN-E | M | 45 | 741 | 49 |  | Not Detected |
|  | HN-F | M | 49 | 493 | 51 |  | Not Detected |
|  | HN-G | M | 36 | 708 | 45 |  | Not Detected |
|  | HN-H | M | 58 | 1438 | 64 |  | Not Detected |
|  | HN-I | M | 47 | 465 | 25 |  | Not Detected |
|  | HN-J | M | 51 | 780 | 27 |  | Not Detected |
|  | HN-K | M | 38 | 885 | 39 |  | Not Detected |
|  | HN-L | M | 29 | 750 | 35 |  | Not Detected |
| **Elite Controllers** | EC-A | M | 56 | 720 | 28 |  | < 40 (37) |
|  | EC-B | M | 42 | 1277 | 48 |  | < 40 (14) |
|  | EC-C | F | 57 | 831 | 40 |  | Not Detected |
|  | EC-D | F | 51 | 1144 | 42 |  | Not Detected |
|  | EC-E | M | 46 | 709 | 44 |  | < 40 (23) |
|  | EC-F | M | 39 | 1018 | 51 |  | Not Detected |
|  | EC-G | M | 41 | 673 | 35 |  | Not Detected |
|  | EC-H | M | 42 | 1326 | 49 |  | Not Detected |
|  | EC-I | M | 58 | 913 | 40 |  | < 40 (37) |
|  | EC-J | M | 57 | 566 | 34 |  | Not Detected |
|  | EC-K | M | 40 | 1204 | 37 |  | < 40 (12) |
|  | EC-L | M | 76 | 925 | 36 |  | < 40 (12) |
| **ART Suppressed** | AS-A | M | 51 | 1054 | 34 | ABC/3TC,LPV/r | Not Detected |
|  | AS-B | M | 46 | 844 | 45 | ABC/3TC,ATV,RTV | Not Detected |
|  | AS-C | M | 55 | 577 | 27 | DDI,3TC,NVP | Not Detected |
|  | AS-D | M | 49 | 1263 | 35 | FTC/TDF,LPV/r | < 40 (17) |
|  | AS-E | M | 71 | 1118 | 29 | EFV/TDF/FTC | Not Detected |
|  | AS-F | M | 41 | 744 | 39 | ABC/3TC,RGV | < 40 (14) |
|  | AS-G | F | 50 | 497 | 27 | FTC/TDF,ATV,RTV | Not Detected |
|  | AS-H | F | 43 | 1036 | 32 | FTC/TDF,ATV,RTV | Not Detected |
|  | AS-I | M | 53 | 878 | 39 | FTC/TDF,ATV,RTV | < 40 (16) |
|  | AS-J | M | 55 | 678 | 25 | ABC/3TC,ATV,RGV | < 40 (29) |
|  | AS-K | M | 51 | 843 | 43 | FTC/TDF,ATV,RTV | Not Detected |
|  | AS-L | M | 57 | 979 | 47 | ETV,RTV,DRV,RGV | Not Detected |
| **Non-controllers** | NC-A | F | 52 | 551 | 24 |  | 62315 |
|  | NC-B | M | 52 | 497 | 22 |  | 11023 |
|  | NC-C | M | 20 | 501 | 23 |  | 89965 |
|  | NC-D | M | 46 | 473 | 16 |  | 39719 |
|  | NC-E | M | 27 | 414 | 16 |  | 43463 |
|  | NC-F | F | 42 | 492 | 34 |  | 7015 |
|  | NC-G | M | 55 | 402 | 34 |  | 79418 |
|  | NC-H | M | 42 | 503 | 32 |  | 7408 |
|  | NC-I | M | 32 | 269 | 20 |  | 21604 |
|  | NC-J | M | 41 | 434 | 21 |  | 48131 |
|  | NC-K | M | 52 | 252 | 33 |  | 18559 |
|  | NC-L | M | 33 | 938 | 32 |  | 10515 |

**Additional file Table 2.** P values for inter-disease state gene expression comparisons.

|  | **HN^1^** | **HN** | **HN** | **EC** | **EC** | **AS** |
| --- | --- | --- | --- | --- | --- | --- |
|  | **vs** | **vs** | **vs** | **Vs** | **vs** | **vs** |
|  | **EC^2^** | **AS^3^** | **NC^4^** | **AS** | **NC** | **NC** |
| **APOBEC3A** | 0.765 | 0.533 | 0.863 | 0.873 | 0.629 | 0.458 |
| **APOBEC3B** | 0.665 | 0.138 | 0.0082 | 0.330 | 0.0257 | 0.0496 |
| **APOBEC3C** | 0.130 | 0.603 | 0.066 | 0.022 | 0.575 | 0.0129 |
| **APOBEC3D** | 0.096 | 0.796 | 0.0325 | 0.0295 | 0.452 | 0.0098 |
| **APOBEC3F** | 0.139 | 0.723 | 0.0093 | 0.065 | 0.270 | 0.0027 |
| **APOBEC3G** | 0.086 | 0.938 | 0.194 | 0.056 | 0.796 | 0.166 |
| **APOBEC3H** | 0.555 | 0.546 | 0.129 | 0.906 | 0.341 | 0.231 |
| **BST2** | 0.115 | 0.802 | 0.0156 | 0.161 | 0.168 | 0.0206 |
| **CDKN1A (P21)** | 0.740 | 0.738 | 0.150 | 0.954 | 0.551 | 0.378 |
| **CTR9** | 0.757 | 0.063 | 0.633 | 0.0283 | 0.410 | 0.087 |
| **EIF2AK2** | 0.392 | 0.847 | 0.0003 | 0.329 | 0.0001 | 0.001 |
| **HERC5** | 0.112 | 0.370 | 0.059 | 0.495 | 0.0001 | 0.0025 |
| **IFITM1** | 0.093 | 0.818 | 0.0002 | 0.161 | 0.007 | 0.0011 |
| **IFITM2** | 0.630 | 0.790 | 0.058 | 0.490 | 0.136 | 0.0484 |
| **IFITM3** | 0.385 | 0.200 | 0.0091 | 0.586 | 0.0298 | 0.092 |
| **ISG15** | 0.818 | 0.331 | 0.0007 | 0.255 | 0.0003 | 0.0246 |
| **MOV10** | 0.538 | 0.371 | 0.013 | 0.182 | 0.134 | 0.0015 |
| **PAF1** | 0.688 | 0.318 | 0.433 | 0.152 | 0.701 | 0.065 |
| **RNASEL** | 0.432 | 0.532 | 0.997 | 0.184 | 0.406 | 0.507 |
| **RSAD2** | 0.614 | 0.674 | 0.0014 | 0.239 | 0.0001 | 0.0023 |
| **RTF1** | 0.099 | 0.076 | 0.991 | 0.128 | 0.996 | 0.056 |
| **SAMHD1** | 0.603 | 0.413 | 0.062 | 0.690 | 0.059 | 0.090 |
| **SLFN11** | 0.436 | 0.002 | 0.0046 | 0.0235 | 0.0481 | 0.632 |
| **TRIM11** | 0.774 | 0.221 | 0.328 | 0.303 | 0.211 | 0.0498 |
| **TRIM14** | 0.155 | 0.878 | 0.0032 | 0.186 | 0.0402 | 0.0037 |
| **TRIM19 (PML)** | 0.296 | 0.588 | 0.0105 | 0.116 | 0.058 | 0.004 |
| **TRIM21** | 0.673 | 0.583 | 0.169 | 0.347 | 0.300 | 0.079 |
| **TRIM22** | 0.566 | 0.391 | 0.0035 | 0.758 | 0.0001 | 0.0001* |
| **TRIM26** | 0.691 | 0.0263 | 0.865 | 0.0378 | 0.583 | 0.026 |
| **TRIM28** | 0.697 | 0.292 | 0.664 | 0.208 | 0.456 | 0.521 |
| **TRIM32** | 0.815 | 0.0159 | 0.899 | 0.0438 | 0.746 | 0.036 |
| **TRIM5** | 0.169 | 0.425 | 0.004 | 0.368 | 0.0359 | 0.0023 |

**^1^** HN: HIV negative controls

**^2^** EC: Elite controllers.

**^3^** AS: ART suppressed

**^4^** NC: non-controllers
